# Acute Chagas disease in the state of Pará, Amazon Region: is it increasing?

**DOI:** 10.1590/0074-02760170298

**Published:** 2018-05-07

**Authors:** Valéria Regina Cavalcante dos Santos, Juliana de Meis, Wilson Savino, Jorge Alberto Azevedo Andrade, José Ricardo dos Santos Vieira, José Rodrigues Coura, Angela Cristina Verissimo Junqueira

**Affiliations:** 1 Secretaria de Estado de Saúde do Pará, Belém, PA, Brasil; 2 Fundação Oswaldo Cruz-Fiocruz, Instituto Oswaldo Cruz, Laboratório de Doenças Parasitárias, Rio de Janeiro, RJ, Brasil; 3 Fundação Oswaldo Cruz-Fiocruz, Instituto Oswaldo Cruz, Laboratório de Pesquisas sobre o Timo, Rio de Janeiro, RJ, Brasil; 4 Fundação Oswaldo Cruz-Fiocruz, Instituto Nacional de Ciência e Tecnologia em Neuroimunomodulação, Rio de Janeiro, RJ, Brasil; 5 Universidade Federal do Pará, Instituto de Ciências Biológicas, Belém, PA, Brasil

**Keywords:** acute Chagas disease, oral transmission, disease surveillance, SINAN

## Abstract

Acute Chagas disease (ACD) has a distinct epidemiological profile in the Amazon Region, with cases and outbreaks of *Trypanosoma cruzi* infection being possibly related to the ingestion of contaminated food. Data on ACD in the state of Pará retrieved from 2000 to 2016 from the Brazilian Notifiable Diseases Information System (SINAN) were evaluated. During this period, 2,030 of the 16,807 reported cases were confirmed, with a higher incidence between the months of August and December, thus characterising a seasonal pattern of acute infection, and coinciding with the higher production of “açaí”, one fruit likely involved in the oral transmission of the disease. Evaluation of the absolute numbers of confirmed ACD cases secondary to oral infection suggests that infection through this route increased during the 2010-2016 period, differing from what was recorded in terms of vectorial or other infection routes. These findings point to the need of intensifying strategies to prevent or substantially reduce oral transmission.

Chagas disease, caused by the protozoan parasite *Trypanosoma cruzi* is an endemic infection of chronic evolution and remains a serious public health problem in Brazil and throughout Latin America, particularly in the Amazon Region. According to the bulletin of the Brazilian Ministry of Health, 1,034 cases of orally transmitted acute Chagas disease (ACD) were registered from 2000 to 2013 (MS 2015). Most of these cases of ACD were outbreaks related to the ingestion of *T. cruzi* contaminated food (açaí, bacaba, consumption of wildlife meat such as capybara, armadillo and opossum by rural populations), revealing a peculiar epidemiological profile that, thus far, has not manifested in an expressive form in traditionally endemic areas (Pinto et al. 2008, Nóbrega et al. 2009, Passos et al. 2012, Shikanai-Yasuda and Carvalho 2012, Coura 2015, MS/SVS 2017). Diagnosis of ACD is accomplished by the identification of *T. cruzi* in blood by direct parasitological methods (Junqueira et al. 2010, Dias et al. 2016). Notifications of clinically suspected and confirmed (diagnostic confirmation) cases follows the protocol of the Brazilian Ministry of Health Notification System (SINAN) (SINAN 2007, MS 2016). Gradually implemented during 1993 by the Brazilian Ministry of Health, the SINAN became regulated through the Ministerial Ordinance No. 073, dated 9 March 1998. Since then, notification has been mandatory for municipalities, states and the Federal District to regularly report the closure of investigation into suspicious cases of ACD to the national database within the minimum period of 60 days after notification using the SINAN reporting/investigation form. The objective of this system is to collect and process compulsory notifications of disease nationally, to delineate the history and magnitude of the disease, to facilitate the detection of outbreaks or epidemics, to guide decision-making at the three governmental levels and to serve as a tool for developing strategies and guidelines for prevention and control of ACD (SINAN 2007, MS 2016).

In the present study we evaluated the occurrence and profile of the notified and confirmed cases of ACD through a descriptive study of ACD cases registered in the SINAN from 2000 to 2016 in the municipalities of the state of Pará in the Brazilian Amazon Region. Although the system registered notification of suspected cases without confirmations in 2000 and 2001, from 2002 to 2016, data of both suspected and confirmed cases were captured.

Currently, the SINAN version 5.0 and the updated 5.2 are available to the entire country (SINAN 2007). From 2000 until 2016, according to the Pará state SINAN, 16,807 notifications of suspected cases from 130 of the 144 municipalities were registered. However, from 2002 to 2016, the system registered 2,030 confirmed ACD cases in 81 municipalities of the same state. Accordingly, an average of 135 confirmed cases per year in the state [standard deviation (SD) = 99.8; median = 114 cases] [95% confidence interval (CI) = 80.0-190.6] were recorded from 2002-2016. The year with the fewest confirmed cases was 2005 with five cases (0.2% of the total), and 2016 showed the largest number, reaching 327 confirmed cases (16.1% of the total). From 1996 to 1999, 75 were confirmed ACD cases, and from 2000 to 2001 there were 50 confirmed ACD cases, records computed by Secretariat of Health Surveillance of the Brazilian Health Ministry, adding a total, in the period from 1996 to 2016 of 2,155 confirmed ACD cases (Fig. 1).

**Fig. 1:**
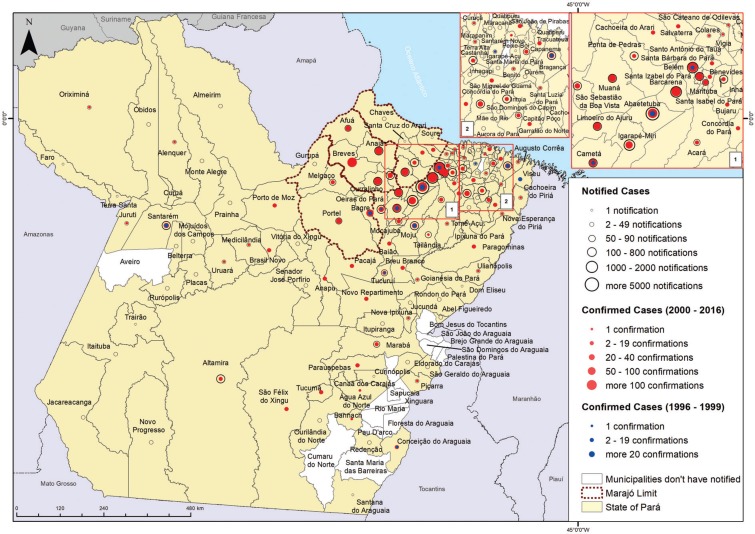
distribution of acute Chagas disease cases during the period 1996-2016, in the state of Pará, Brazil. Database: Brazilian Ministry of Health Notification System (SINAN) 1996-1999. Pará state SINAN 2000-2016.

We also calculated the percentage of autochthonous cases notified (suspected ACD) and confirmed (by laboratory identification of *T. cruzi*) in each municipality. The percentage of confirmed cases in relation to notified cases was 12.1% (Table). The municipality of Abaetetuba had the largest number of cases reported (4,205; 25.0% of notifications) followed by Belém (1,713; 10.2%) and Barcarena (1,611; 9.6%). However, the analysis of confirmed cases in relation to cases notified by municipalities showed that Barcarena and Abaetetuba had low percentages of confirmation (8.8% and 7.4%, respectively), whereas Bagre, Anajás and Portel had high percentages (83.7%, 54.9% and 44.9%, respectively). Regarding other municipalities, we observed that Capanema, Marambá, Bujaru, Pacajá, Baião, Conceição do Araguaia and São Miguel do Guamá reported 54, 51, 49, 45, 44, 36 and 34 cases, respectively, with confirmed cases ranging from 2 to 10 cases.

**TABLE:** Notified and confirmed autochthonous cases of acute Chagas disease in the state of Pará (Brazil) from 2002 to 2016

Municipalities	Notifications	%	Confirmations	%	% C/N
Abaetetuba	4205	25.0	311	15.3	7. 4
Belém	171 3	10.2	3 41	16 . 8	19.9
Barcarena	1611	9.6	14 2	7. 0	8.8
Igarapé-Miri	1596	9.5	99	4.9	6.2
Cametá	928	5.5	77	3.8	8.3
Moju	7 31	4.3	35	1.7	4.8
Ponta de Pedras	607	3.6	18	0.9	3.0
Muaná	545	3.2	50	2.5	9.2
Breves	504	3.0	212	10.4	42.1
Ananindeua	400	2.4	85	4.2	21.3
Santarém	320	1.9	33	1.6	10.3
Santa Isabel do Pará	262	1.6	6	0.3	2.3
São Sebastião da Boa Vista	247	1.5	40	2.0	16.2
Limoeiro do Ajuru	226	1.3	41	2.0	18.1
Castanhal	219	1.3	17	0.8	7.8
São Domingos do Capim	203	1.2	21	1.0	10.3
Curralinho	166	1.0	55	2.7	33.1
Irituia	153	0.9	11	0.5	7.2
Oeiras do Pará	138	0.8	28	1.4	20.3
Altamira	108	0.6	5	0.2	4.6
Bragança	105	0.6	30	1.5	28.6
Anajás	102	0.6	56	2.8	54.9
Afuá	89	0.5	26	1.3	29.2
Acará	88	0.5	17	0.8	19.3
Melgaço	82	0.5	18	0.9	22.0
Tucuruí	80	0.5	12	0.6	15.0
Marituba	71	0.4	20	1.0	28.2
Tailândia	70	0.4	1	0.0	1.4
Portel	69	0.4	31	1.5	44.9
Mocajuba	64	0.4	4	0.2	6.3
Capitão Poço	50	0.3	2	0.1	4.0
Bagre	41	0.2	36	1.8	87.8
Other municipalities	1000	5.9	150	7.4	15.0
Total	16,793	100	2,030	100	12.1

C/N: % of confirmed cases in relation to notified cases.

Source: Pará state SINAN.

We further analysed whether or not the numbers of suspected and confirmed cases of ACD were increasing. For that, Pearson linear correlation analysis was used to estimate tendency of increase/decrease of cases, considering a 95% level of significance (p < 0.05), using the software Bioestat version 5 (Brazil). We found a significant increase in the number of notified suspected cases (p < 0.0001) and confirmed cases (p = 0.0001) throughout the study period. However, the percentage of confirmed cases in relation to those notified decreased significantly during the same period (p = 0.012). Hypotheses for the difference between the notified and confirmed cases include an improved performance of the State's Chagas disease programme since its implementation through publicity campaigns and training of inspectors for sanitary surveillance, alerting the public to the risks of oral contamination by açaí and indicated a greater cooperation by the population with respect to the investigation of the disease. Another hypothesis is the lack of permanence of trained professionals in the municipalities, which makes the identification of the parasite by laboratory diagnosis more difficult.

As demonstrated in Fig. 1, ACD cases were mainly present in the metropolitan region of Belém, Marajó and the north-eastern part of the state. This difference in number of ACD cases may be related to the large population in these regions. According to Brazilian Institute of Geography and Statistics, the production of açaí (*Euterpe oleracea*) in the state of Pará has increased over recent years, with 13,000 producers being responsible for 54% of the national production, reaching around 800,000 tons per year (IBGE 2015, Tavares and Homma 2015, MAPA 2017). The municipalities of the mesoregion of the northeastern part of Pará, especially Abaetetuba, Igarapé-Miri, Cametá, Moju and Barcarena, as well in the Marajó Island (Portel, Ponta de Pedras and Anajás) stand out with regard to açaí juice production in Pará, supplying the fruit for local or regional metropolitan consumption, and for distribution after pulp pasteurisation to other states as well as for export. In Belém, an estimated 200,000 litres/day are consumed during the harvest period, being the second-most consumed food in the city (Rogez 2000, de Santana et al. 2008, 2012, MAPA 2017). In a recent study on oral ACD by our group (unpublished observations), an epidemiological investigation concerning food consumption revealed that 99% of the 137 patients aged from 18 to 77 years, responded that they consumed açaí in their daily routine (lunch and/or dinner), suggesting that the consumption of juice contaminated by *T. cruzi* is involved and contributes to the appearance of cases of ACD in the state. This hypothesis has been supported by previous studies that have shown the maintenance of the viability and virulence of *T. cruzi* in açaí. These experiments demonstrated that even after refrigerating (4°C) or freezing (-20°C) the pulp of the fruit, *T. cruzi* was still able to promote infection in mice (Rogez 2000, Pérez-Gutiérrez et al. 2006, Passos et al. 2012).

Evaluation of the confirmed ACD cases throughout the year revealed that the largest number of confirmed cases occurred between August and December, indicating a seasonal pattern for the disease. This period coincides with the highest açaí production, concentrated in the second half of the year, with higher yields in the months of September, October, November and December (Rogez 2000, de Santana et al. 2008, 2012). It should be noted that the processing of açaí in Pará is done in an artisanal manner, with extraction of the pulp being accomplished with the aid of a beating or pulping machine. The possibility of contamination is not a recent occurrence since oral transmission of the disease has been recorded in the Amazon Region since the 1960s (Shaw et al. 1969, Dias et al. 2008, Pinto et al. 2008, Nóbrega et al. 2009).

Fig. 2 shows that an increase in cases is expected during the course of each year beginning in July and peaking in September. This distribution corroborates results previously reported by Pinto et al. (2008).

**Fig. 2:**
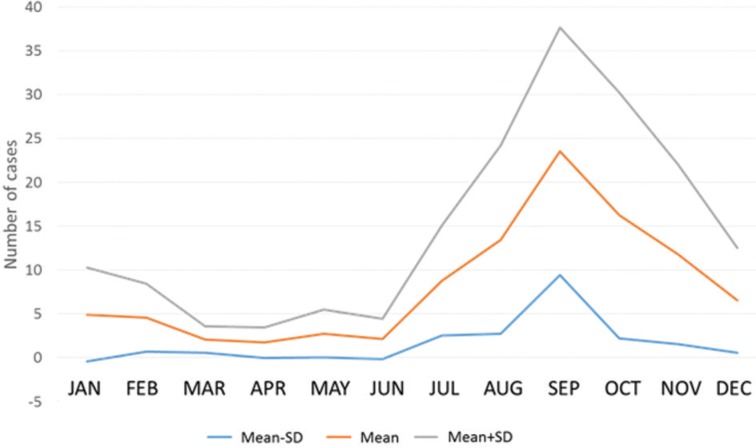
monthly distribution of acute Chagas disease cases confirmed during the period 2002-2016, in the state of Pará, Brazil. The middle curve indicates the mean cases, whereas the top and bottom curves depict one standard deviation. Data were retrieved from the SINAN database in the state, 2016.

The epidemiological profile of the disease presents a scenario with the occurrence of the majority of cases suggestive of oral transmission in the form of an outbreak or in the absence of triatomine bugs in the residences. According to the epidemiological bulletin of 2015 (Ministry of Health), in the period 2000-2013, 812 cases of oral transmission of Chagas disease were confirmed in the state of Pará (MS 2015). These numbers seem to be underestimated, since according to the SINAN state records, from just 2007 to 2013, 851 cases of oral transmission were confirmed in Pará.

The most affected age group corresponded to adults between 18 and 60 years with 69% (38% male and 31% female, respectively) of the cases. The age group 0 to 17 years accounted for 19% (10% male and 9% female, respectively) and over 60 years to 12% (6% male and 6% female, respectively) of the cases.

In the specific records of oral transmission, males were also more affected within each age group, except the age groups of 13-17 years and over 60 years in which females were more affected. Analysis of the locations of the official residence of patients revealed that ACD cases in urban and rural areas numbered 1,020 and 880, respectively. This difference can be attributed to the larger population in the urban area, with 5,197,118 inhabitants compared to the 2,390,960 in the rural population, according to the last national census undertaken in 2010. This finding suggests that the urban area has a larger number of inhabitants who consume açaí on a daily basis. It may also be related to the fact that in the urban area, the diagnosis and active surveillance of cases are conducted better (Pickenhayn et al. 2008, Urdaneta-Morales 2014, Alroy et al. 2015).

We observed that the most prevalent mode of transmission was oral, which accounted for 74.3% of the cases; the remaining 25.7% were attributed to vector pathways (13.7%), ignored (11.2%), transplacental (0.2%), accidental (0.2%) and others (0.4%). It is interesting to note that according to the SINAN, in the state of Pará recording of oral infection commenced in 2007, suggesting an under-reporting in the system before 2007 (Fig. 3). The most frequent clinical manifestations were fever (85-100% of patients), asthenia and facial oedema (60-70%) or the lower limbs (22-47%), and generalised oedema (4-14%). Oedema has been previously associated with the acute phase of oral infection (Pinto et al. 2008, Shikanai-Yasuda and Carvalho 2012, Alarcón de Noya et al. 2015, Souza and Povoa 2016). A chagoma due to inoculation, which is the signal of entry of the classical vector pathway, was present in only 1-2% of the records. Acute chagasic patients associated with an outbreak due to oral transmission in Venezuela revealed symptoms such as fever, headache, myalgia, and oedema, with a mean of 9 ± 4 symptoms per patient (range of 5-16) and 44% lethality. The most common symptoms in fatal cases were hepatomegaly (100%), myocarditis (75%), pericardial effusion (50%) and cardiomegaly (25%) (Añez et al. 2016).

**Fig. 3:**
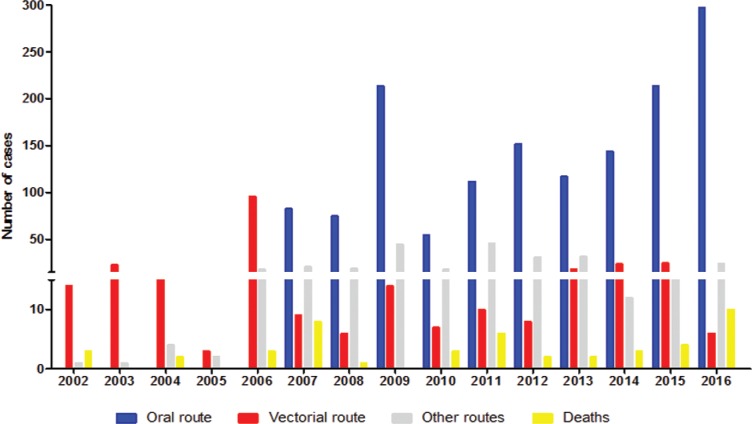
distribution of acute Chagas disease cases regarding the putative source of infection and deaths, during the period 2002-2016, in the state of Pará, Brazil.

Regarding the evolution of the cases investigated in the present study, 48 deaths were recorded of all cases (2,030) with a lethality rate of 2% and peaks in 2007, 2011 and 2016. Twenty-seven deaths (56.25%) were attributed to oral infection, 9 (18.75%) to vector infection and 12 (25%) were of unknown infection routes (Fig. 3). One reason for this number of deaths may be the late diagnosis of infection and cardiac compromise, including acute chagasic myocarditis. In this regard, since cardiomyopathy is an important cause of death in patients with Chagas disease, and with high morbidity, early diagnosis and prompt treatment is of paramount importance and may alter the clinical course of the disease (Pinto et al. 2008, MS/SVS 2012, Souza and Povoa 2016).

The state of Pará possesses an açaí production chain that involves approximately 300,000 people. The fruit arrives daily in Belém to be sold, and during the period of harvest, 471,000 litres of açaí are sold at around 3,000 points of sale (CONTECC 2015, SEDAP 2015). The Brazilian National Health Surveillance Agency (ANVISA) prepared a training programme for Sanitary Surveillance Bodies, açaí beaters and the whole population. This document highlights the recommendations of the Pan American Health Organisation for the inclusion of Chagas disease as a foodborne disease and the implementation of efficient heat treatment protocols where the main focus is to eliminate *T. cruzi* from açaí juice (ANVISA 2008). According to the Decree No. 326, dated 20 January 2012, which establishes good practice and procedures for the handling of açaí, and the recommendations of the Secretary of Health Surveillance for the prevention, control and clinical management of cases due to oral contamination, one of the effective strategies for preventing the disease is the “bleaching” or “whitening” of the açaí, which is accomplished by thermal shock (MS/SVS 2012). We observed no evidence of a decrease in the number of cases over the years, which pose an increasing challenge for public health. Early diagnosis and prompt notification of ACD cases is thus indispensable. Moreover, an epidemiological investigation must be conducted in order to establish the actual risk factors. It is essential, that healthcare professionals understand that the absence of records regarding specific laboratory tests for ACD and inadequate completion of the reporting and investigation forms compromise the closure of cases. Obtaining quality data is an essential condition for the health system to detect failures and to formulate guidelines for interventions to effectively control the disease. Identification of the appropriate tools is highly relevant to ensure that information used by managers facilitates the development of public policies and aids in the planning of actions so that SINAN data provide information as close as possible to the reality experienced by the population to support the planning of control measures. Based on the increase in the number of cases over the course of 15 years, which may be due to population growth or other risk factors attributed to the consumption of food contaminated with metacyclic trypomastigote forms of *T. cruzi*, we suggest that prevention and control measures established by ANVISA and the Sanitary Surveillance in the state of Pará must be intensified, especially in the municipalities that have been reporting cases of ACD annually. Among the various measures we highlight are good agricultural practices by extractivists and producers to protect açaí from contamination during primary production, and the “bleaching” or “whitening” of the fruits as a routine practice of quality control of the artisanal açaí consumed by the population in Pará, since industrialised production utilises pasteurised pulp (ANVISA 2008).

In conclusion, even with a huge difference between the number of confirmed cases of ACD and the notification (suspected) cases reported, there was a clear increase in numbers of ACD cases during the last decade. Taking this into account, campaigns to prevent oral transmission in the state of Pará urgently need to be intensified to prevent or substantially reduce oral transmission.
